# Shear stress regulation of nanoparticle uptake in vascular endothelial cells

**DOI:** 10.1093/rb/rbad047

**Published:** 2023-05-02

**Authors:** Hongping Zhang, Ziqiu Hu, Jinxuan Wang, Jianxiong Xu, Xiangxiu Wang, Guangchao Zang, Juhui Qiu, Guixue Wang

**Affiliations:** Key Laboratory for Biorheological Science and Technology of Ministry of Education, State and Local Joint Engineering Laboratory for Vascular Implants, Bioengineering College of Chongqing University, Chongqing 400030, China; Key Laboratory for Biorheological Science and Technology of Ministry of Education, State and Local Joint Engineering Laboratory for Vascular Implants, Bioengineering College of Chongqing University, Chongqing 400030, China; Key Laboratory for Biorheological Science and Technology of Ministry of Education, State and Local Joint Engineering Laboratory for Vascular Implants, Bioengineering College of Chongqing University, Chongqing 400030, China; Key Laboratory for Biorheological Science and Technology of Ministry of Education, State and Local Joint Engineering Laboratory for Vascular Implants, Bioengineering College of Chongqing University, Chongqing 400030, China; Key Laboratory for Biorheological Science and Technology of Ministry of Education, State and Local Joint Engineering Laboratory for Vascular Implants, Bioengineering College of Chongqing University, Chongqing 400030, China; Lab Teaching & Management Center, Chongqing Medical University, Chongqing 400016, China; Key Laboratory for Biorheological Science and Technology of Ministry of Education, State and Local Joint Engineering Laboratory for Vascular Implants, Bioengineering College of Chongqing University, Chongqing 400030, China; JinFeng Laboratory, Chongqing 401329, China; Key Laboratory for Biorheological Science and Technology of Ministry of Education, State and Local Joint Engineering Laboratory for Vascular Implants, Bioengineering College of Chongqing University, Chongqing 400030, China; JinFeng Laboratory, Chongqing 401329, China

**Keywords:** shear stress, nanoparticle uptake, endothelial cell, clathrin, caveolin

## Abstract

Nanoparticles (NPs) hold tremendous targeting potential in cardiovascular disease and regenerative medicine, and exciting clinical applications are coming into light. Vascular endothelial cells (ECs) exposure to different magnitudes and patterns of shear stress (SS) generated by blood flow could engulf NPs in the blood. However, an unclear understanding of the role of SS on NP uptake is hindering the progress in improving the targeting of NP therapies. Here, the temporal and spatial distribution of SS in vascular ECs and the effect of different SS on NP uptake in ECs are highlighted. The mechanism of SS affecting NP uptake through regulating the cellular ROS level, endothelial glycocalyx and membrane fluidity is summarized, and the molecules containing clathrin and caveolin in the engulfment process are elucidated. SS targeting NPs are expected to overcome the current bottlenecks and change the field of targeting nanomedicine. This assessment on how SS affects the cell uptake of NPs and the marginalization of NPs in blood vessels could guide future research in cell biology and vascular targeting drugs.

## Graphical Abstract

**Figure:**
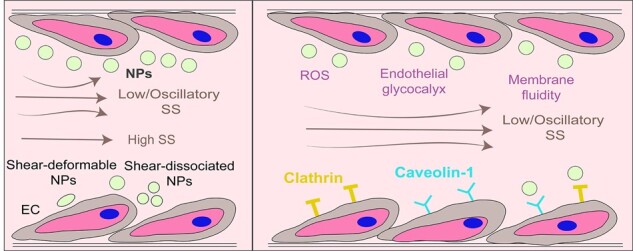

## Introduction

Nano-therapeutics have the potential to provide cures for humanity’s most vulnerable diseases, such as cardiovascular disease and cancer. Nanoparticles (NPs) have been proven as a promising material for biomedical systems due to their favorable biodistribution, stability solubility, specific target and sustained release kinetics [1–5]. The core advantage of NPs is that they could be efficiently engulfed by cells as they have the unique characteristics of small size and high surface area to volume ratio. Currently, NPs have varieties of potential applications in the biomedical field, such as drug delivery [6–11], imaging [12–16] and anticancer therapy [17–23].

The cellular mechanical microenvironment is an essential regulator of cell homeostasis and cellular function. While numerous barriers must be overcome to reach the target site, in most cases, the therapeutic effect for a nanomedicine at its target site is ultimately governed by the efficiency of NPs to enter the cell, which involves the NP uptake by cells and the location of NPs in blood vessels. Blood flow could alter the uptake of NPs [24–26] and affect their transportation *in vivo*, thereby influencing the overall uptake of NPs [27–29]. In addition, intravenously injected nanomedicine could experience different magnitudes of shear stress (SS) in blood vessels. An understanding of how SS affects cellular NP uptake could be beneficial to controlling the dosage and toxicity of NPs to obtain remarkable therapeutic effects of nanomedicines.

In this review, the latest advances in the development of cellular NP uptake affected by SS were summarized, and the marginalization of NPs in blood vessels was generalized. First, the effects of different SS on NP uptake by endothelial cells (ECs) were highlighted for the first time. Second, the biomechanical mechanism on cellular NP uptake was summarized. Third, the longitudinal distribution of NPs with different sizes and stiffness in blood vessels was generalized.

## SS parameter

SS refers to the force per unit area created when a tangential force (blood flow) acts on the endothelium surface [30], which is related to blood viscosity and blood flow velocity at the vessel wall. The blood flow produces pulsatile flow and oscillatory flow. In general, the SS generated by pulsatile flow could be divided into low SS (LSS) and high SS (HSS) in accordance with magnitude. LSS could promote the occurrence of AS, and HSS could protect the blood vessels. In addition, the oscillatory SS (OSS) generated by oscillatory flow could promote the occurrence of AS. Notably, the magnitude of SS *in vivo* varies with blood vessel location. For instance, the magnitudes of SS range from 1 to 6 dyne/cm^2^ in the venous vessels and from 10 to 70 dyne/cm^2^ in the arterial vessels [31]. Blood flow velocities vary in the atherosclerosis (AS) plaque area and the bifurcation and bending of arteries [32].

**Figure 1.**
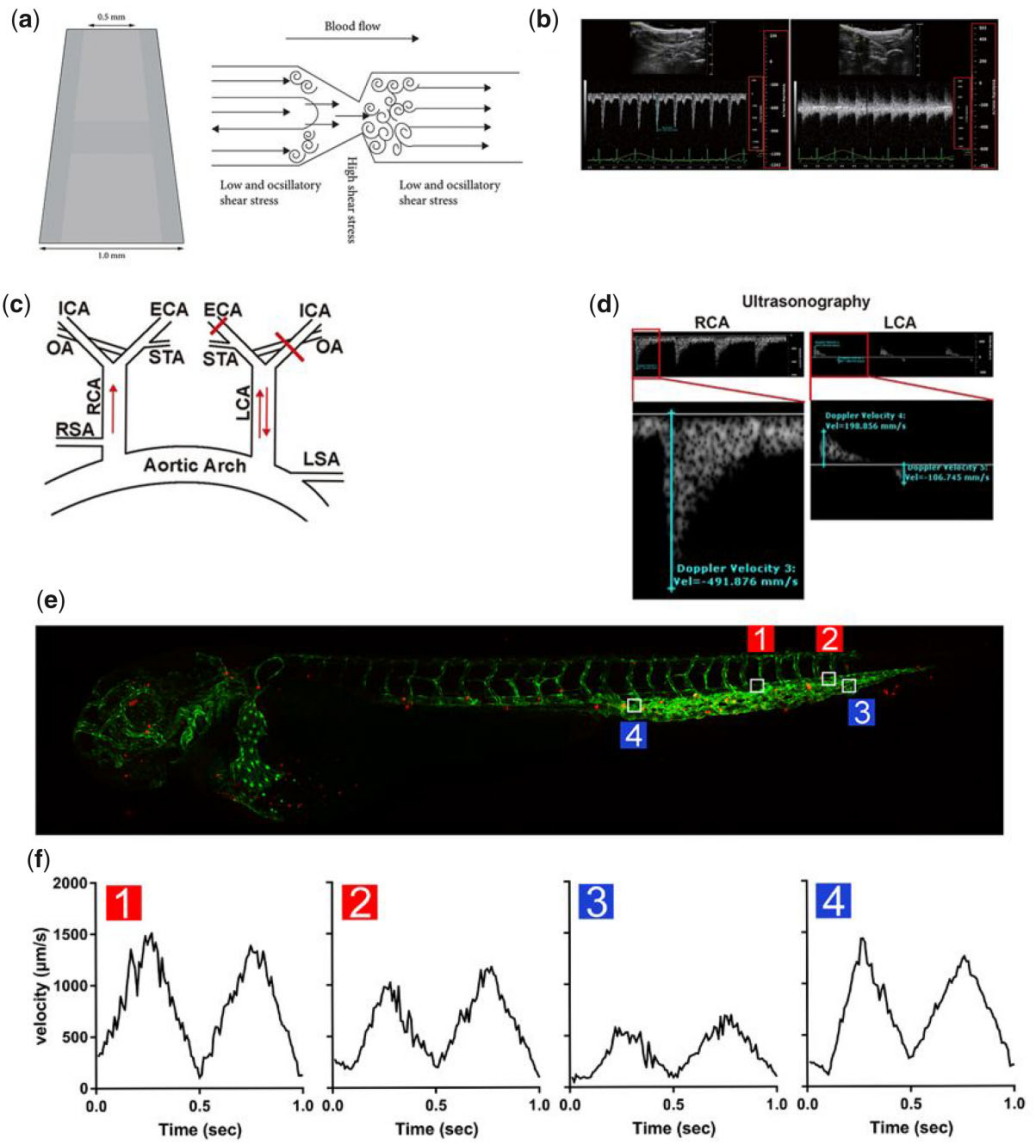
SS model *in vivo*. (**a**) Carotid artery constriction model using conical silicone cast. Panel (**b**) is the flow velocity of (a). Reproduced with permission from Ref. [38]. Copyright 2019, Hindawi. (**c**) Partial carotid arteries ligation model using sterile surgical instruments. RCA, right carotid arteries; LCA, left carotid arteries; ICA, internal carotid artery; ECA, external carotid artery; OA, occipital artery; STA, superior thyroid artery. Panel (**d**) is the flow velocity of (c). Reproduced with permission from Ref. [25]. Copyright 2018, Springer Nature Switzerland AG. (**e**) Model diagram of zebrafish. (**f**) Blood flow velocities in four different blood flow regions. Reproduced with permission from Ref. [37]. Copyright 2021, Elsevier B.V.

Normal SS (NSS) is unidirectional in relatively straight arterial segments. However, blood flow could produce LSS and/or OSS in regions of irregularly shaped vessels. LSS typically occurs in curved internal areas and the upstream of stenosis [33], whereas OSS commonly presents in the downstream of stenosis, bifurcated sidewalls and branch points [34]. Various models, including *in vivo* and *in vitro* [35], have been recently constructed to discover the effects of SS on cell fate. In a carotid artery constriction model, LSS and OSS could be generated at the upstream and downstream of the narrow area, whereas HSS could be generated at the narrow area (Fig. 1a and b). In a mouse model of partial carotid artery ligation, the right carotid artery without ligation could generate NSS, whereas the left carotid artery with partial ligation could generate OSS (Fig. 1c and d). In addition, the blood flow velocity at different positions of zebra fish tail varies greatly (Fig. 1e and f). Furthermore, zebrafish have been widely used in toxicological and mechanical studies due to their unique advantages [36, 37]. Most of these *in vivo* models are only used to accelerate or control the induction of AS, ignoring the magnitudes of SS. In addition, SS may vary widely among individual animals. Hence, the accuracy of extrapolating data from previous studies is insufficient, and the magnitude of SS for each animal needs to be calculated.

## NP engulfment in ECs under different SS

ECs are generally affected by the SS generated from blood flow, which is a key regulator in NP uptake. They are mechanoresponsive [39, 40] as they initially respond to changes in the blood flow, and many EC surface mechanosensors [41, 42] have been identified. Similar to static condition [43], various parameters could affect the cellular uptake of NPs under mechanical conditions, such as internalization time and concentration of NPs, the properties of NPs, surface modification, the magnitude of SS and fluid patterns (Table 1).

**Table 1.** Factors affecting cellular uptake of NPs under SS

SS (dyne/cm^2^)	Parameter	Cell type	Summary	References
5	1, 12 and 24 h	HUVEC	NP uptake increases as the uptake time increases	[37]
5	5–75 μg/ml	HUVEC	NP uptake increases as NP concentration increases	[37]
10	800 × 100 × 100 nm^3^ rods 400 × 100 × 100 nm^3^ rods	HUVEC	Longer NPs are more likely to be engulfed	[44]
10	325 nm diameter × 100 nm high disks 220 nm diameter × 100 nm high disks	HUVEC	Larger NPs are more likely to be engulfed	[44]
≈2	UEA-1 modification	HUVEC	Modification with UEA-1, which binds to HUVEC, could promote NP uptake	[45]
5–25	5, 12 and 25 dyne/cm^2^	HUVEC	LSS promotes NP uptake	[37]
12 0.5 ± 4	Flow patterns	HUVEC	OSS promotes NP uptake	[37]

### LSS enhances engulfment

**Figure 2.**
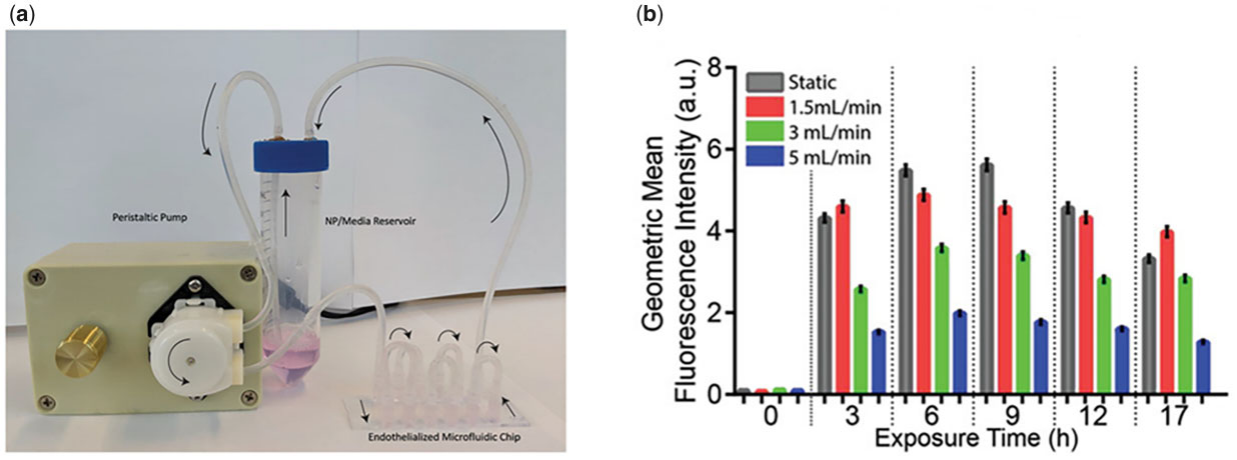
Using a microfluidic system to demonstrate that LSS promotes uptake of NPs by ECs. (**a**) Schematic of the microfluidic system. (**b**) Fluorescence intensity of NP uptake by ECs at different time points with different flow rates. Reproduced with permission from Ref. [45]. Copyright 2020, WILEY-VCH Verlag GmbH & Co. KGaA, Weinheim.

SS and flow velocity are the crucial regulators in the progression of AS [46–50]. Numerous studies have shown that LSS-induced EC activation and dysfunction are an essential process in the development of AS [51–53]. Cells exposed on LSS could promote NP uptake with a faster rate [54] and significantly increase cellular uptake of NPs [55]. Compared with static state, LSS (0.5 dyne/cm^2^) increased the uptake of cationic polystyrene NPs by mouse pancreatic ECs [56]. The surface modifications of NPs could affect cellular uptake under dynamic conditions [57–59]. By using a microfluidic system, Chen’s group found that when human umbilical vein endothelial cells (HUVECs) engulfed NPs under different SS for 3 h, LSS could promote NP uptake (Fig. 2), and the uptake of NPs by HUVECs increased when gold NPs were modified with Ulex Europaeus Agglutinin-1 lectin, which could bind to HUVECs [45].

Extracellular vesicles (EVs) are membranous vesicles produced by cells [60–63]. As EVs have the ability to transfer bioactive components and cross biological barriers, they are gradually innovating as a potential therapeutic vehicle for various drugs [64–68]. The authors’ group utilized red blood cell EVs (RBCEVs) to investigate the effects of SS on cellular uptake [37]. Compared with 12 dyne/cm^2^ (NSS), HUVECs exposed to 5 dyne/cm^2^ (LSS) could increase the vesicle uptake by HUVECs. Simultaneously, HUVECs barely internalized RBCEVs under HSS.

### OSS boosts engulfment

AS mainly occurs in ECs exposed to OSS with focal occurrence, such as arterial bifurcations and bends. The authors’ previous work showed that LSS and OSS may increase the uptake of low-density lipoprotein (LDL) by ECs [25, 69]. Moreover, NPs tended to accumulate in the areas of blood vessel bifurcations and blood flow oscillations, especially near the inner wall of blood vessels [39, 70].

**Figure 3.**
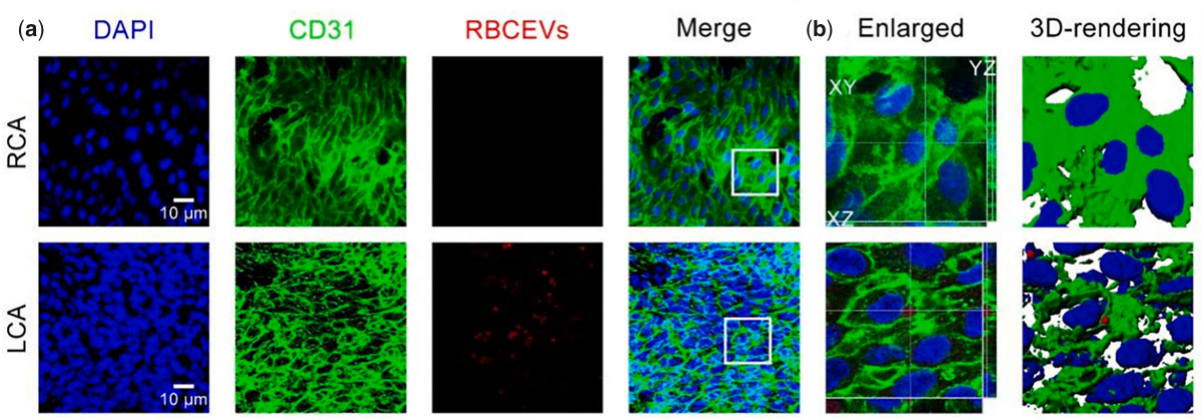
OSS promotes the uptake of RBCEVs by ECs in carotid artery ligation mice. (**a**) En face immunofluorescence images. OSS increased the uptake of RBCEVs by ECs. (**b**) Imaris 3D rendering of (a). Reproduced with permission from Ref. [37]. Copyright 2021, Elsevier B.V.

Inflammation and macrophage infiltration are indicators of AS pathogenesis [71–73]. Therefore, macrophage membrane-coated biomimetic nanomedicines have been served to treat AS, and they could accumulate in regions with OSS, such as the aortic arch [74, 75]. Meanwhile, drug delivery systems coated by RBCEVs are also an excellent strategy for the treatment of AS [76]. Erythrocyte membrane-coated nanodrugs could accumulate to the regions of AS plaques with OSS, and RBCEVs could be engulfed by ECs in the regions of OSS in carotid artery ligation mice (Fig. 3) [37]. In addition, HUVEC is more inclined to engulf mesoporous silica nanodisk than mesoporous silica nanosphere under OSS [77].

### Shear-deformable NPs and shear-dissociated NP aggregates alleviate NP uptake inhibited by HSS

SS could increase to 1000 dyne/cm^2^ due to bleeding and cardiovascular disease accompanied with oscillatory flow [78, 79]. Compared with the traditional drug delivery systems, the nanodrug delivery systems controlled by SS have many benefits, such as high efficiency, low side effects and easy modeling [80–82]. However, compared with LSS and OSS, HSS decreases the uptake of NPs by cells [37]. Therefore, designing NPs that could target the sites with HSS is necessary. Shear-deformable NPs [83] and NP aggregates (NPAs) [84] were introduced in 2012, resulting in the appearance of nanomaterials specifically designed for shear-triggered release. Shear-deformable NPs are one of the main types of carriers for shear-triggered drug. They release the cargo during specific SS. At present, shear-deformable NPs, including spherical liposomes [85, 86], lenticular liposomes [83, 87], nanogels [88, 89] and micellar hydrogels [90, 91], have been successfully prepared.

**Figure 4.**
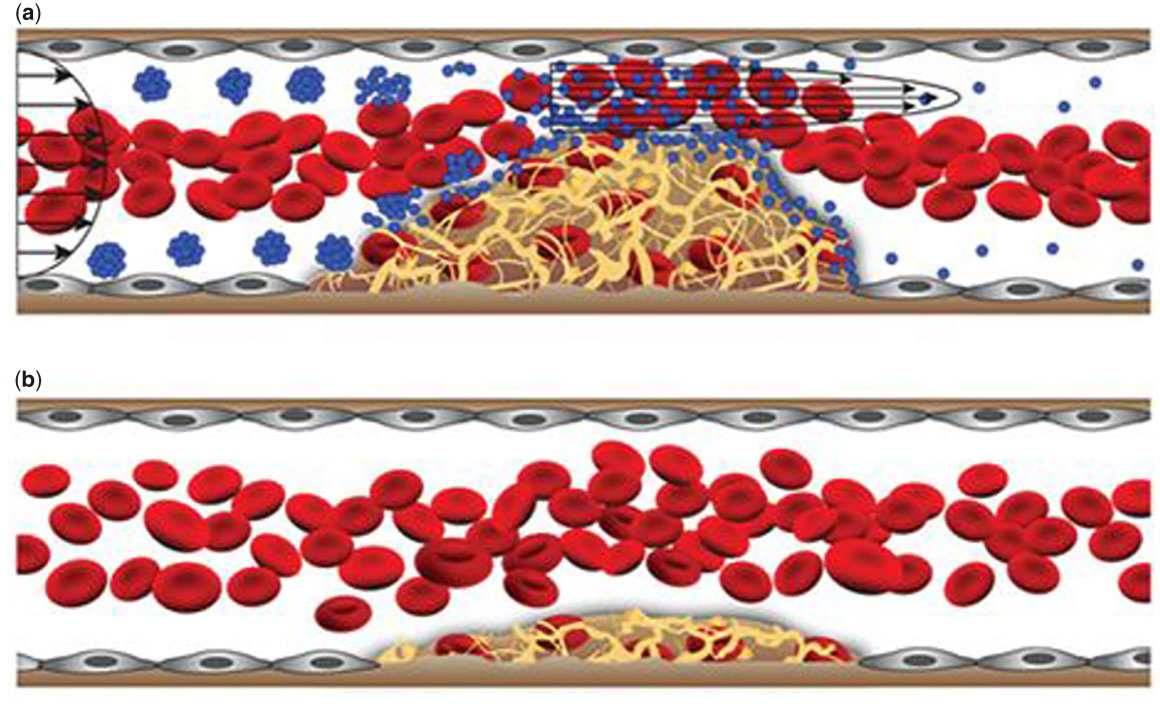
Shear targeting of a thrombolytic drug in arterial thrombosis model by using shear-activated nanotherapeutics (SA-NTs). (**a**) SA-NTs disintegrate into single NP due to local HSS at thrombus site. (**b**) Accumulation of tPA-coated NPs and binding to the clot at the occlusion site progressively dissolve the obstruction. Reproduced with permission from Ref. [84]. Copyright 2021, American Association for the Advancement of Science.

Instead of deforming to deliver nanodrugs, NPAs are disintegrated into single NPs to deliver drugs. Korin’s group constructed tissue-type plasminogen activator (tPA) nanomedicine coated by PLGA to dissolve thrombus [84]. Under NSS, the nanomedicine formed microscale aggregates via hydrophobic interactions but disintegrated into single NPs when the SS was > 100 dyne/cm^2^. Single NPs could bind to the vessel wall more easily as they experience less resistance, enabling localized drug delivery at sites of HSS (Fig. 4). NPAs could target occlusive blood vessels by autonomous dispersion and gather at tissue sites by depolymerizing during injection and re-aggregating at the damaged tissue sites [92]. The aggregation of nanomedicines depends on the NP quality, fractal dimension, coating and crosslinker properties [93, 94]. The macromolecules adsorbed on the surface of NPs also have effects on NP aggregation [95, 96]. NPAs with specific SS sensitivity could be obtained by changing the parameters of nanomaterials, and a reversible aggregation system has been successfully exploited [78, 84].

In summary, LSS and OSS may promote ECs to engulf NPs, independent of the nature of the material itself. However, HSS could decrease NP uptake because it reduces the contact and residence time of NPs with ECs. Therefore, shear-deformable NPs and NPAs released in response to HSS have been developed to target HSS sites.

## Potential mechanism for NP engulfment under SS

Understanding the specific mechanism on how SS affects cell uptake could also contribute to developing newly therapeutic strategies. SS affects not only the level of intracellular ROS [97, 98], endothelial glycocalyx [99] and membrane fluidity [100] but also the level of protein expression [101, 102].

### Cellular oxidative stress in regulation of SS-induced uptake

Oxidative stress in the vascular wall is a risk factor for the development of AS. Treatment of ECs with ox-LDL could activate NOX4 to increase vascular oxidative stress [103]. Meanwhile, OSS could also induce oxidative stress in ECs [38, 39]. LSS-induced oxidative stress is primarily accomplished through activation of the mammalian target of rapamycin complex 1 and subsequent phosphorylation of eNOS-Thr495. The oxidative stress and apoptosis in HUVECs induced by LSS were attenuated by rapamycin targeting the mTORC2 signal and its downstream sestrin [104].

**Figure 5.**
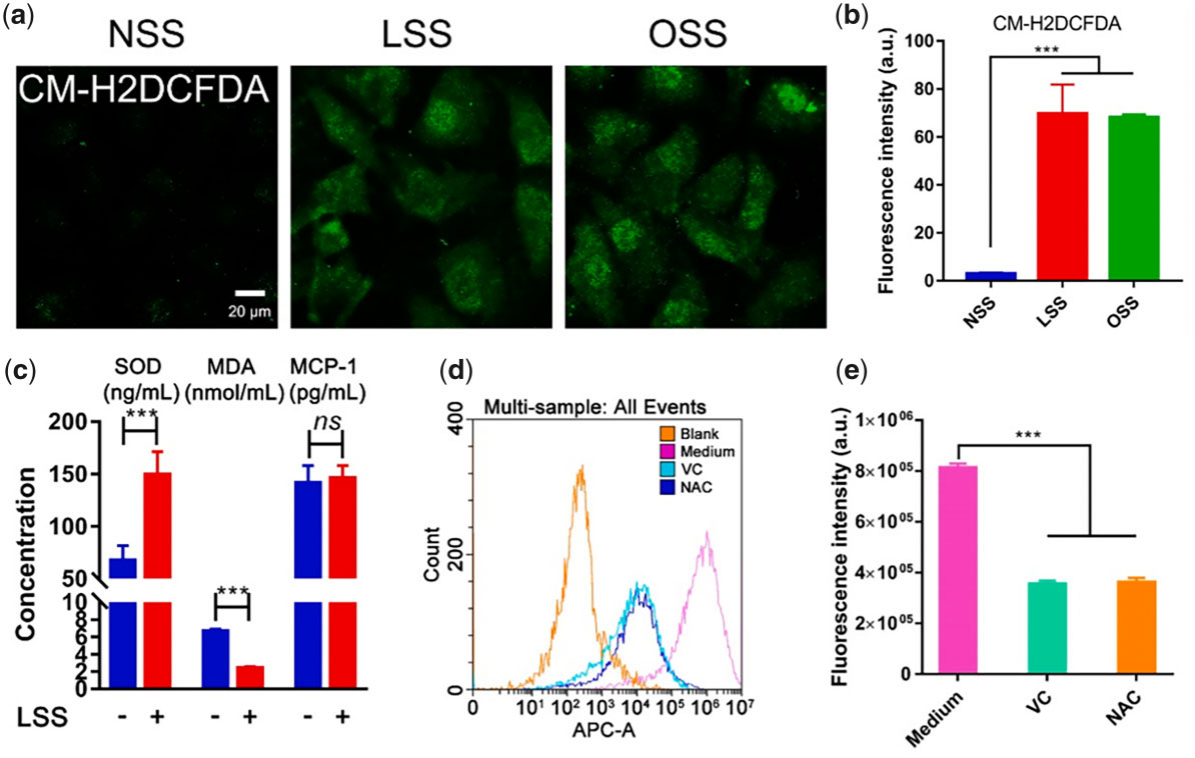
Oxidative stress is the regulator in LSS-induced RBCEVs uptake by ECs. (**a**) and (**b**) LSS and OSS could increase ROS generation. (**c**) SOD, MDA and MCP-1 levels were detected by ELISA. (**d**) and (**e**) Cellular uptake of RBCEVs was reduced after antioxidant pretreatment. Reproduced with permission from Ref. [37]. Copyright 2021, Elsevier B.V.

A previous study found that OSS/LSS-induced oxidative stress is a regulator of RBCEV uptake by ECs [37]. LSS led to an increase in superoxide dismutase (SOD) protein level and a decrease in malondialdehyde (MDA) protein level, whereas the expression of monocyte chemoattractant protein 1 did not significantly change, demonstrating that LSS could rapidly induce oxidative stress. The intracellular ROS induced by LSS was inhibited with two general antioxidants, l-ascorbic acid (VC) and *N*-acety-l-cysteine. The ROS levels in ECs were alleviated, and the uptake efficiency of RBCEVs by ECs was significantly reduced. Therefore, LSS has been proven to induce oxidative stress in ECs, thereby increasing the uptake of RBCEVs (Fig. 5).

### Endothelial glycocalyx in regulation of SS-induced uptake

Endothelial glycocalyx is a general term for the polysaccharide protein complex covering the surface of vascular ECs. It is mainly composed of bound glycosaminoglycans, anchored proteoglycans, binding glycoproteins and adsorbed soluble molecules. In healthy individuals, endothelial glycocalyx plays a key role in maintaining the normal function of ECs and provides a vascular permeability barrier. However, in patients with hypertension, diabetes, kidney disease and other diseases related to increased risk of AS, endothelial glycocalyx is damaged and falls off.

Endothelial glycocalyx plays an important role in the response of ECs to SS. On the one hand, endothelial glycocalyx is necessary for EC cytoskeleton to respond to SS. On the other hand, endothelial glycocalyx injury may change the role of HSS from protecting blood vessels to accelerating the rupture of AS plaque. Compared with normal areas, the coverage and thickness of endothelial glycocalyx in plaque areas were significantly reduced in ApoE^–/–^ mice [105]. Treated ECs with 12 dyne/cm^2^ or HepIII enzyme could lead to endothelial glycocalyx damage. The uptake of ultrafine gold NPs by ECs increased, while endothelial glycocalyx was damaged [106].

### Membrane fluidity in regulation of SS-induced uptake

Membrane fluidity is one of the basic characteristics of membrane structure, which mainly refers to the movement state of membrane fatty acid chain and membrane protein [107]. Under the stimulation of SS, the lipid order of EC membrane changed from liquid order to liquid disorder, and the fluidity of cell membrane increased [108]. When the membrane fluidity increased, the NP uptake by ECs increased [109].

### Transmembrane protein in regulation of SS-induced uptake

#### Clathrin in regulation of SS-induced uptake

Clathrin-mediated endocytosis (CME) exists in many mammalian cells, and it is the primary pathway for cells to obtain nutrients. CME begins with the accumulation of clathrin on the inner surface of the plasma membrane, and the formation of clathrin-coated pits begins with the interaction of various proteins, such as dynein, on the outer surface of the cell. Subsequently, under the action of actin, the pit rapidly invaginates to form a clathrin-coated vesicle. Chlorpromazine could inhibit adaptor protein 2, and chloroquine could influence the function of clathrin and clathrin-coated vesicles. These two chemical inhibitors could be used to inhibit CME [110]. Furthermore, dynamin-2 and clathrin could be knocked out to investigate CME.

One crucial regulator of NP uptake is clathrin. Albumin uptake is unaffected under static while the primary cilia in cells are removed. However, the increase in albumin uptake induced by fluid shear stress (FSS) is nearly completely abolished [111]. Moreover, the uptake of albumin was significantly reduced in OK cells after treatment with the clathrin inhibitor chlorpromazine and the dynamin inhibitor Dyngo-4a. FSS could affect the expression of clathrin, which affects the endocytosis of NPs. Ertl’s group found that when the SS was <4 dyne/cm^2^, the amount of polystyrene engulfed by ECs gradually increased with the increase in SS magnitude [112]. The expression levels of clathrin under different SS indicated that the increased uptake was caused by shear-dependent increase in clathrin on the cell surface. Subsequently, Xu’s group also obtained similar results, i.e. FSS upregulated the expression of clathrin in HK-2 cells, resulting in higher uptake of NPs under flow conditions [113].

#### Caveolae-associated protein in regulation of SS-induced uptake

Caveolae-mediated endocytosis is involved in the transcellular transport of NPs in ECs. Caveolae connect to the plasma membrane by tiny necks, forming ∼60 nm spherical pits in diameter. So far, no chemical inhibitor could specifically inhibit this process. In general, interfering or knocking out caveolin components, such as Caveolin-1 (Cav-1), Caveolin-3 or Cavin 1, could be applied to investigate this endocytosis process.

The fluid pattern may affect the expression of Cav-1. Turbulent flow reduces the expression of Cav-1 compared with laminar flow [114]. SS mediated by caveolae could activate YAP/TAZ in response to mechanical stimulation [115]. Therefore, caveolae are crucial mechanosensors [116, 117]. Cav-1 also plays a significant role in various cancer-related processes, such as tumor growth and metastasis [118–120].

**Figure 6.**
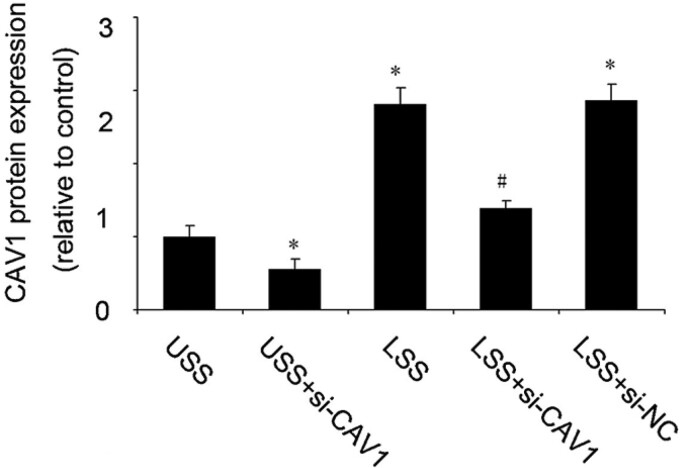
SS regulates caveolae-associated protein expression levels. LSS upregulated the protein expression of Cav-1 compared with undisturbed shear stress (USS). Reproduced with permission [122]. Copyright 2016, Elsevier Ltd.

Compared with negligible caveolae in capillary ECs, the numerous caveolae in arteriole ECs are an essential element for neurovascular coupling of arteriole ECs [121]. Caveolae could respond to FSS [122], which has effect on uptake of lipids (Fig. 6). Compared with that in LDL-receptor-deficient (*Ldlr^–/–^*) mice, the level of lipid accumulation at the aortic arch and plaque area in *Ldlr^–/–^ Cav1^–/–^* mice was markedly reduced [123]. Apart from SS, cyclic stretches increase the cellular uptake of NPs and convert the phagocytic pathway from CME under static conditions to caveolae-mediated pathway under stretched state [124]. Harder substrate stiffness also upregulates Cav-1 expression, which increases NP uptake by cells [125]. In addition, stiff NPs enter cells through the clathrin-mediated pathway, whereas soft NPs prefer to enter cells via the caveolae-mediated pathway [126].

## NP distribution under SS environment

The NP spatial distribution in the blood vessel is an additional parameter that could influence the uptake of NPs. The distribution of NPs depends on the flow velocity of the bloodstream, which may affect their adhesion to cells [27, 28]. The accumulation of NPs is also influenced by blood characteristics, such as hematocrit [127]. At constant hematocrit, drug accumulation reaches a maximum when wall SS is minimal. Flow velocity is a crucial parameter for the distribution of NPs. A slower flow velocity increases the migration of circulating NPs to the vascular wall, thus promoting the interaction between NPs and ECs [128]. Rinker investigated the effect of charge and fluid velocity on the distribution of quantum dots (QDs) and found that positively charged QD were easier to aggregate in ECs. In a zebrafish model, the QDs accumulated more in the venous vessels, with an average velocity of 300 μm/s, than in the dorsal artery, with an average velocity of 600 μm/s [129]. Similarly, the authors’ group found that RBCEVs tended to accumulate in the venous plexus with lower flow velocity in a zebrafish model [37]. Other studies qualitatively demonstrated that NPs preferentially accumulated in low-flow vascular regions of zebrafish embryos and mice [130–132]. In general, in comparison with high-velocity blood flow, low-velocity blood flow may increase the contact time of NPs with cells, which may be a reason for NPs inclining to accumulate and distribute at the region with low-velocity blood flow.

**Figure 7.**
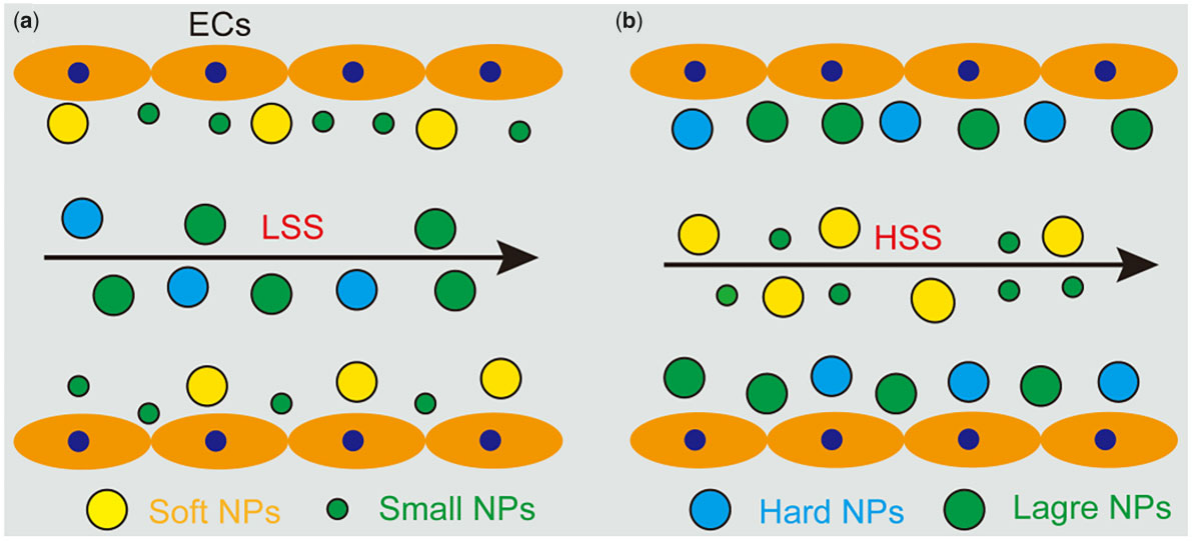
Longitudinal distribution of NPs with different sizes and stiffness in blood vessels under different SS. (**a**) Soft and small NPs are more likely to be marginalized under LSS. (**b**) Hard and large NPs are easier to deposit on the surface of blood vessels under HSS.

Despite NPs tending to accumulate in regions with lower blood flow velocity, various NPs have been developed to respond to areas of HSS, thereby increasing the efficacy of treatment [133]. Surprisingly, the uptake of NPs could be reversed by SS. For instance, hard NPs are more prone to marginate at HSS than soft NPs easily deposited under LSS [134, 135]. In other examples, small NPs are more likely to be marginalized at LSS, whereas large NPs preferably marginalize at HSS [136, 137] (Fig. 7). In constant blood flow, small spherical and rod-shaped NPs incline to distribute in the red blood cell core, resulting in less contact with ECs [138]. Notably, larger particles with a size of 1–2 µm readily distribute in the acellular layer to interact with the endothelium. Rod-shaped particles may exhibit enhanced marginalization due to increased drift forces and tumbling motions in the flow.

## Conclusion and perspectives

For nanodrug delivery, understanding the relationship among SS, NPs and cells is crucial. Herein, an overview of the current understanding of how SS affects NP uptake and distribution was provided, in the hope of stimulating research aimed at designing NPs targeting for different SS. Compared with HSS, NPs tend to distribute at the LSS/OSS area, and the longitudinal distribution of NPs with diverse characteristics could be reversed by SS. Furthermore, SS increases NP uptake by increasing the level of intracellular ROS and membrane fluidity, upregulating the expression of clathrin and Cav-1, and downregulating the expression of endothelial glycocalyx. In addition, designing NPs to target blood vessel walls is a key process for successful drug delivery in cardiovascular diseases. Improved understanding of these features could potentially help harness cell biomechanics mechanisms for more efficient nanodrug delivery.

Despite substantial advances in the understanding of SS on NP uptake by cells, several challenges remain that limit the widespread clinical application of NPs that are truly released in response to SS. One of the major issues is how to distinguish the relationship between distribution and uptake regulated by SS. In this review, the uptake of NPs at locations with different SS and the longitudinal distribution of NPs in blood vessels were highlighted. However, the distribution of NPs on the cell membrane surface was counted as NP uptake in numerous studies, which could greatly affect the actual uptake. The cellular uptake of NPs requires NPs to enter the cytoplasm rather than just distributing in the blood vessels or adhering to the cell surface. The other issue is that the effect of SS on intracellular uptake of NPs was almost all verified by HUVECs. However, the aortic ECs at the aortic arch and bifurcation also experience OSS. Therefore, using aortic ECs to verify the uptake of NPs in cardiovascular disease seems more appropriate. Finally, whether NPs could be precisely designed to target therapeutic delivery via SS is still unclear, because most of the current research is processed in the laboratory.

With the development of drug delivery, the central dogma is an increasing requirement for the mechanical mechanism of engulfment. ECs are mechanically responsive, and their gene expression is regulated by the local SS [139], which may affect its tendency to engulf NPs. Therefore, the interplay between blood flow and cellular homeostasis is coordinated by cell phagocytosis of NPs.
